# Single Nucleotide Polymorphisms of the *GJB2* and *GJB6* Genes Are Associated with Autosomal Recessive Nonsyndromic Hearing Loss

**DOI:** 10.1155/2015/318727

**Published:** 2015-05-17

**Authors:** Ana Paula Grillo, Flávia Marcorin de Oliveira, Gabriela Queila de Carvalho, Ruan Felipe Vieira Medrano, Sueli Matilde da Silva-Costa, Edi Lúcia Sartorato, Camila Andréa de Oliveira

**Affiliations:** ^1^Laboratory of Genetics and Molecular Biology, Graduate Program in Biomedical Sciences, Centro Universitário Hermínio Ometto (UNIARARAS), Avenida Maximiliano Barutto No. 500, Jardim Universitário, 13607339 Araras, SP, Brazil; ^2^School of Biology, Centro Universitário Hermínio Ometto (UNIARARAS), 13607339 Araras, SP, Brazil; ^3^School of Biomedicine, Centro Universitário Hermínio Ometto (UNIARARAS), 13607339 Araras, SP, Brazil; ^4^Center of Molecular Biology and Genetic Engineering (CBMEG), Molecular Biology Laboratory, State University of Campinas (UNICAMP), 13083-970 Campinas, SP, Brazil

## Abstract

Single nucleotide polymorphisms (SNPs) are important markers in many studies that link DNA sequence variations to phenotypic changes; such studies are expected to advance the understanding of human physiology and elucidate the molecular basis of diseases. The *DFNB1* locus, which contains the *GJB2* and *GJB6* genes, plays a key role in nonsyndromic hearing loss. Previous studies have identified important mutations in this locus, but the contribution of SNPs in the genes has not yet been much investigated. The aim of this study was to investigate the association of nine polymorphisms located within the *DFNB1* locus with the occurrence of autosomal recessive nonsyndromic hearing loss (ARNSHL). The SNPs rs3751385 (C/T), rs7994748 (C/T), rs7329857 (C/T), rs7987302 (G/A), rs7322538 (G/A), rs9315400 (C/T), rs877098 (C/T), rs945369 (A/C), and rs7333214 (T/G) were genotyped in 122 deaf patients and 132 healthy controls using allele-specific PCR. There were statistically significant differences between patients and controls, in terms of allelic frequencies in the SNPs rs3751385, rs7994748, rs7329857, rs7987302, rs945369, and rs7333214 (*P* < 0.05). No significant differences between the two groups were observed for rs7322538, rs9315400, and rs877098. Our results suggest that SNPs present in the *GJB2* and *GJB6* genes may have an influence on ARNSHL in humans.

## 1. Introduction

Hearing loss (HL) is a common congenital sensory disorder worldwide, affecting almost 600 million people. Approximately 2 to 6 children in 1,000 are affected by severe hearing loss at birth [1] or during early childhood. This is defined as prelingual deafness, with about half of cases attributable to genetic causes. Furthermore, many cases of late onset progressive HL also have a genetic origin, in addition to progressive HL associated with ageing [2]. According to the Hereditary Hearing Loss (HHL) homepage (http://hereditaryhearingloss.org/), over 140 loci for nonsyndromic HHL have been mapped, together with approximately 80 genes, and more than 1000 mutations have been identified in humans, making it one of the most genetically heterogeneous traits. However, in most cases, genetic hearing loss is a monogenic disorder. Biallelic mutations in 47 different genes have been reported for autosomal recessive nonsyndromic hearing loss (ARNSHL), which in many populations accounts for 80% of families with this type of deafness [3].

The occurrence of ARNSHL has been related to the* DFNB1* locus (deafness, autosomal recessive 1) (OMIM 220290) at chromosome 13q11-q12, which contains two genes associated with hearing loss:* GJB2* (gap junction protein, beta-2) (OMIM 121011) and* GJB6* (gap junction protein, beta-6) (OMIM 604418), which codify the proteins encoding connexins 26 (Cx26) [4] and 30 (Cx30) [5], respectively. Mutations in these genes are the most frequent causes of ARNSHL in most populations worldwide, sometimes accounting for up to 50% of cases [6].

Direct intercellular communication is mainly mediated by gap junction channels, which in vertebrates are formed by members of the connexin protein family. Connexins are transmembrane proteins that regulate electrical signals and the passage between neighboring cells of ions, small biological molecules (<1000 Da) including sugars, nucleotides, and amino acids, secondary messengers such as Ca^2+^, cyclic AMP, and inositol triphosphate, and metabolic precursors [7]. Cochlear gap junctions, especially connexins Cx26 and Cx30, have been implicated in the maintenance of K^+^ homeostasis in the inner ear [8]. Cx26 and Cx30, the two most abundantly expressed gap junction proteins in the cochlea, are coexpressed as heteromeric connexons in nonsensory cells of the organ of Corti as well as in cells of the spiral ligament and stria vascularis [9]. Extensive genetic studies have been conducted to identify mutations in the* DFNB1* locus; however, information is lacking concerning the potential association between nonsyndromic hearing loss and SNPs of the* GJB2* and* GJB6* genes [10].

Single nucleotide polymorphisms (SNPs) are the most abundant genetic markers at a specific location in the genome, occurring at a frequency of more than 1% in the human population [11]. The International HapMap Project has characterized over 3.1 million human SNPs, indicating a SNP density of approximately one per kilobase [12]. SNPs are of great interest in medical and pharmacological studies of disease susceptibility and drug response. They also provide powerful tools for a variety of medical genetic studies [13].

In view of all these observations, in an attempt to identify polymorphisms related to autosomal recessive nonsyndromic hearing loss, this study investigated the frequency of SNPs in the* GJB2* and* GJB6* genes by means of a case-control association study.

## 2. Materials and Methods

### 2.1. Ethics Statement

Written informed consent forms were obtained from all the participating subjects or from their parents. The study was approved by the Ethics Committee at Centro Universitário Hermínio Ometto de Araras (UNIARARAS), under protocol number 744/2010.

### 2.2. Study Subjects

The study involved 122 unrelated newborn patients of both genders (69 males and 53 females) with moderate to profound ARNSHL, together with 132 normal controls (CTL). The patients were from the Hearing and Language Stimulation Therapy Association, in Jundiaí (SP, Brazil). This institution is concerned with auditory and communication disorders, and since 2001 it has developed a universal hearing screening program. The majority of subjects studied were classified as Caucasian; however, many Brazilians have unique mixtures of Amerindian, European, and African ancestries in their genomic mosaic [14].

All patients underwent molecular analysis of the coding region and part of exon 1 and the flanking donor splicing site of* GJB2* and the two deletions affecting the* GJB6* gene (del(*GJB6*-D13S1830) and del(*GJB6*-D13S1854)). Also the 1555A>G, 827A>G, and 1494C>T mitochondrial mutations in the* MTRNR1* gene and the 74555A>G mutation in the* MTTS1* gene were analyzed. Following this screening, all individuals with an identified molecular cause for ARNSHL were excluded from the study. These previous analyses were performed at the Center of Molecular Biology and Genetic Engineering (CBMEG), Human Molecular Genetics Laboratory, State University of Campinas (UNICAMP), Campinas, SP, Brazil.

The normal controls were recruited from amongst the employees of UNIARARAS. In order to exclude the influence of inherited susceptibility factors, information about the control individuals was collected using questionnaires containing items concerning general health, medical conditions, and hereditary factors. Subjects with histories of head injury, otological disease, other diseases that could affect hearing, and previous or present treatment with ototoxic substances were excluded.

### 2.3. SNP Selection

Several SNPs covering the* DFNB1* locus were analyzed, seven of these (rs3751385, rs7994748, rs7329857, rs7987302, rs877098, rs945369, and rs7333214) were selected from the HapMap database (available at http://www.hapmap.org/), and SNP genotyping data from the YRI population were downloaded into Haploview [15]. Only SNPs that passed quality control criteria (call rate ≥ 95%, minor allele frequency (MAF) ≥ 0.05, and Hardy-Weinberg disequilibrium *P* > 0.01) were included in the genetic analysis. Two other SNPs (rs7322538 and rs9315400) were selected based on previous reports of association with deafness phenotypes [10]. Nine SNPs were analyzed, four in the* GJB2* gene and five in the* GJB6* gene. A summary of the selected SNPs is provided in Table 1.

### 2.4. Genotyping

Genomic DNA was extracted from whole blood leukocytes using the standard phenol–chloroform method, as described previously [16]. For genotyping, all the DNA samples were normalized to a concentration of 50 ng/*μ*L.

The SNP genotypes were determined by allele-specific PCR (AS-PCR) amplification. For analysis of allelic variants, two forward primers were designed, with the 3′ base of each primer matching only one of the biallelic SNP bases to be evaluated. Incorporation of a primer mismatch at the second or third base from the 3′ end of the primer has been shown to enhance the specificity of the PCR by further destabilizing the extension of the doubly mismatched primer [17–19]. A common reverse primer (COM) was designed downstream of the polymorphic site. Control primers were also used for SNP coamplification of a portion of the human amelogenin (*AMELX*) gene [20]. These primers were therefore used as internal amplification controls. The sequences of the primers used in this study are listed in Table 2.

Approximately 10% of samples, randomly selected, were regenotyped for cross-validation by restriction fragment length polymorphism PCR (RFLP-PCR) using restriction enzyme (New England Biolabs Inc., USA) (see Table 2) or by direct sequencing of PCR products using ABI BigDye Terminator, with analysis using an ABI PRISM 3700 DNA sequencer (Applied Biosystems, Foster City, USA). No inconsistencies were observed.

### 2.5. AS-PCR Amplification and Electrophoresis

The PCR procedures were performed using a 30 *μ*L reaction volume containing 50 ng of template DNA, 0.5–0.7 pmol of each forward and reverse primer, 0.2 pmol of each control primer, 170 *μ*M dNTP, an appropriate concentration of MgCl_2_ (Table 3), 0.01% (v/v) BSA, 1X reaction buffer (50 mM KCl, 20 mM Tris-HCl, pH 8.4), and 1 U* Taq* polymerase (Invitrogen, Itapevi, SP, Brazil). The samples were incubated at 95°C for 5 min, followed by 30 cycles of 1 min denaturation at 95°C, 1 min annealing (the times and annealing temperatures for different PCRs are described in Table 3), 1 min extension at 72°C, and a final extension at 72°C for 10 min. The amplicons (Table 3) were separated by electrophoresis in 1.5% agarose gel stained with ethidium bromide and were visualized and photographed using the Syngene G:BOX gel documentation system.

### 2.6. Statistical Analysis

The Pearson goodness of fit *χ* ^2^ test was used to assess deviation from Hardy-Weinberg equilibrium (HWE). Differences in genotype and allele frequencies between the study groups were compared by the chi-square test and/or Fisher's exact test. Association between the SNPs and the risk of autosomal recessive nonsyndromic hearing loss was analyzed using the binary logistic regression test. Risk was expressed as odds ratio (OR) with 95% confidence intervals (CI). An OR > 1.0 was used as the cutoff for the baseline of risk-associated SNPs, and the baseline for risk-lowering SNPs was OR < 1.0. The Bonferroni post hoc test was used to compare multiple groups, using the definition *P* value (single tests) × number of tests. The minor allele frequency (MAF) for the study was calculated, and a *χ* ^2^ test was performed to determine whether there was a significant difference between the MAF value obtained here and that in the public MAF database (NCBI dbSNP Build 132). All the statistical analyses were conducted using GraphPad Prism 5.0 software. A *P* value < 0.05 was taken as statistically significant.

## 3. Results

**Figure 1:**
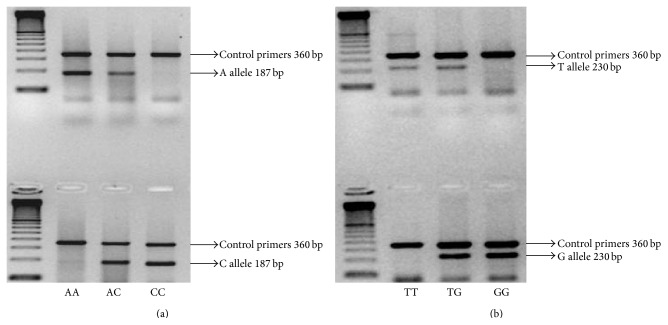
Electrophoretic patterns observed on AS-PCR analysis of the SNPs rs945369 (a) and rs7333214 (b). DNA ladder molecular weight marker (100 bp) was run in the first lane as labeled. The photo shows all three genotypes obtained from study individuals seen on the ethidium bromide-stained 1.5% agarose gel. AA: homozygote for the allele A; AC: heterozygote for the alleles A and C; CC: homozygote for the allele C (a). TT: homozygote for the allele T; TG: heterozygote for the alleles T and G; GG: homozygote for the allele G (b).

A total of 254 individuals (132 in the control group and 122 in the patient group) were genotyped for 9 SNPs in the* DFNB1* locus, using AS-PCR.  Figure 1 shows a representative AS-PCR gel corresponding to the SNPs rs945369 and rs7333214. Significant deviations from Hardy-Weinberg equilibrium were detected in the genotype frequencies of both groups. HWE *P* values for all the studied SNPs are summarized in Table 4. Analysis of the results indicated that the minor allele frequencies of the significant SNPs were in accordance with the information in the public database, although different frequencies were found for two of the SNPs, rs7994748 (MAF = 0.173) and rs7987302 (MAF = 0.026) (Table 5).

Out of the nine SNPs included in the study, six (66.7%) were found to be significantly associated with ARNSHL. Four of these (rs3751385, rs7994748, rs7329857, and rs7987302) were found in the* GJB2* gene and two (rs945369 and rs7333214) in the* GJB6* gene (Table 6). No significant associations were found for the other three SNPs (33.3%) genotyped. Three of the four significant SNPs of the* GJB2* gene (rs3751385, rs7994748, and rs7329857) and one SNP of the* GJB6* gene (rs7333214) remained statistically significant after application of the Bonferroni correction for multiple testing (×9, *P* values of 1.011 × 10^−12^, 0.036, 3.478 × 10^−4^, and 0.0027, resp.). These four SNPs were therefore of sufficient interest to warrant further investigation. As revealed by the odds ratio (OR), four of the six SNPs were associated with a high risk of ARNSHL, with OR > 1, while the remaining two were associated with a decreased susceptibility to ARNSHL (OR < 1). The rs7329857 SNP of the* GJB2* gene was associated with the highest risk of ARNSHL, with OR of 11.70.

Considering the risk-increasing SNPs, the rs3751385 (C/T) T allele (*GJB2* gene) was identified in 90 out of 122 ARNSHL cases (74%), with 40 in heterozygous and 50 in homozygous genotypes. Likewise, the T allele of rs7994748 (C/T) was present in 92% of the cases (10 in heterozygous and 102 in homozygous genotypes). Other risk-associated SNPs in the* GJB2* gene were only observed to occur in heterozygous genotypes, and these included 20 cases (16%) for rs7329857 (C/T) and 10 cases (8%) for rs7987302 (G/A). The presence of rs945369 (A/C) in the* GJB6* gene was observed to be associated with decreased risk of ARNSHL, with 38 of the patients heterozygous (31%) and 23 of the cases (19%) CC homozygous. A genetic association of SNP rs7333214 (T/G) with ARNSHL was identified in 94% of the patients, with 61 heterozygous and 54 GG homozygous genotypes. The results of the association analysis are summarized in Table 6.

## 4. Discussion

Genome-wide association studies (GWAS) have successfully identified numerous loci that influence disease risk. Such techniques have been proved to offer powerful approaches for the screening of genes involved in complex diseases [21], including hearing loss, which occurs in around one per 1,000 newborns on average [22].

The current study evaluated the relationships between polymorphisms in the* GJB2* and* GJB6* genes and autosomal recessive nonsyndromic hearing loss in a sample of the Brazilian population. Evidence was found that SNPs in both genes were significantly associated with ARNSHL. Data for 122 patients and 132 controls indicated that* GJB2* rs3751385 (C/T) was associated with hearing loss susceptibility, in agreement with previously published research [21, 23, 24].

The T allele of this SNP (rs3751385) has also been shown to be a risk factor associated with chronic plaque psoriasis in Chinese Han population patients [25]. A similar finding was reported for Chinese patients with psoriatic arthritis [26], possibly because connexin 26 (Cx26) has been found to be highly expressed in psoriasis plaques. Cx26 is widely expressed in most human tissues, including the ectoderm-derived epithelia of the cochlea, cornea, and skin [27].

Wilch et al. [28] previously showed that the expression of* GJB2* and* GJB6* diminished in the presence of the rs7333214 G allele. Conversely, in this study, the presence of the T allele was found to be positively associated with ARNSHL.

Here, to the best of our knowledge, we describe for the first time the association between the rs7994748, rs7329857, rs7987302, and rs945369 SNPs and ARNSHL. The results indicate that these genetic changes may be important determinants of hearing impairment risk in the studied population. The rs7994748 and rs945369 SNPs are present in the intronic regions of the* GJB2* and* GJB6* genes, respectively. It has been shown previously that SNPs present in the noncoding region can modulate gene expression [29]. The rs3751385 and rs7329857 SNPs are located in the 3′UTR region of the* GJB2* gene, while rs7333214 is found in the* GJB6* gene. The 3′UTR region of a gene is usually important for regulation of processes such as transcript cleavage, alternative polyadenylation, and mRNA nuclear export [30]. Mutations in 3′UTR of certain genes have been reported to be associated with several genetic diseases [31–33]. Recently, Ramsebner et al. [34] showed that the rs117685390 C allele in the regulatory region of the human* GJB2* gene could contribute to autosomal recessive nonsyndromic hearing loss.

Our study showed no significant differences between control and hearing loss subjects in terms of the distribution of alleles of the rs7322538, rs9315400, and rs877098 SNPs in the* GJB6* gene. Similarly, no significant differences were detected between noise-induced hearing loss (NIHL) and normal groups when Abreu-Silva and colleagues [35] compared allele and genotype frequencies for the rs877098 SNP. Elsewhere, negative associations of rs7322538 with sporadic hearing impairment were found in the Chinese population [10]. The same study showed a significant difference in allele frequency for rs9315400, most likely due to the different ethnicity.

## 5. Conclusions

The present findings indicate that the rs7322538, rs9315400, and rs877098 SNPs in the* GJB6* gene are not a significant risk factor for the development of ARNSHL in the Brazilian population. However, in contrast to these SNPs, it was found that carriers of the T allele of the SNPs rs3751385 (C/T), rs7994748 (C/T), rs7329857 (C/T), and rs7333214 (T/G) are at increased risk of ARNSHL.

Further studies are required to confirm these findings and to explore the hypothesis that the rs7994748 T, rs7329857 T, rs7987302 A, and rs945369 A alleles could be used as biomarkers for the development of autosomal recessive nonsyndromic hearing loss in Brazilian populations and could be included in assessments of the risk of developing ARNSHL. Finally, we provide important evidence of the association of SNPs in the* GJB2* and* GJB6* genes with hearing loss. The findings of this study contribute to an understanding of the intricate associations and gene interactions involved in hereditary hearing loss.

## Figures and Tables

**Table 1:** Summary of selected SNPs.

Gene	rs number	Position	SNP type	MAF
*GJB2 *	rs3751385	3′ UTR	C/T	T = 0.360
	rs7994748	Intron	C/T	C = 0.481
	rs7329857	3′ UTR	C/T	T = 0.079
	rs7987302	Downstream	G/A	A = 0.098

*GJB6 *	rs7322538	Downstream	G/A	A = 0.214
	rs9315400	Intron	C/T	T = 0.362
	rs877098	Intron	C/T	T = 0.429
	rs945369	Intron	A/C	C = 0.352
	rs7333214	3′ UTR	T/G	T = 0.302

rs number, NCBI reference SNP (rs) number, is an identification tag assigned by NCBI to SNPs [36]. MAF (ref): minor allele frequency information from public database, NCBI dbSNP Build 132; MAF ≥ 0.05.

**Table 2:** Validation methods for each SNP analyzed.

SNP	Gene	Method (enzyme)
rs3751385	*GJB2 *	RFLP-PCR (*Nhe* I)
rs7994748		RFLP-PCR (*Bstx *I)
rs7329857		RFLP-PCR (*Dpn *II)
rs7987302		Direct sequencing

rs7322538	*GJB6 *	Direct sequencing
rs9315400		RFLP-PCR (*Acu *I)
rs877098		RFLP-PCR (*Eco *RI)
rs945369		RFLP-PCR (*Mva *I)
rs7333214		RFLP-PCR (*Mae *II)

**Table 3:** AS-PCR primers and conditions.

Gene	SNP ID (nucleotide change)	Sequence (5′-3′)^*^	Annealing temperature	Mg^2+^	Amplicon size
*GJB2 *	rs3751385 (C/T)	C allele: GCTCAGCTGTCAAGGCTCAGTCTCC T allele: GCTCAGCTGTCAAGGCTCAGTCTCT COM: TTGTCCTCAGAGAAAGAAACAAATGCC	60°C (1 min)	2.5 mM	284 bp
	rs7994748 (C/T)	C allele: GCTGAGAGCTGGGTTCCGTGTC T allele: GCTGAGAGCTGGGTTCCGTGTT COM: AGGGGCTCAGAAGCAGGACG	60°C (1 min)	2.5 mM	435 bp
	rs7329857 (C/T)	C allele: TTTCCCAACACAAAGATTCTGCC T allele: TTTCCCAACACAAAGATTCTGCT COM: CTTACACCAATAACCCCTAACAGCC	58°C (1 min)	2.5 mM	199 bp
	rs7987302 (G/A)	G allele: GGCATATCAGTCTATGGACAATGGGG A allele: GGCATATCAGTCTATGGACAATGGGA COM: AGAGGTTGCAGTGAGCCAAGG	58°C (1 min)	1.7 mM	157 bp

*GJB6 *	rs7322538 (G/A)	G allele: CTAATGCAACTAGGGAAATTCG A allele: CTAATGCAACTAGGGAAATTCA COM: GCAATCTAGTTTTTCCTCATCC	56°C (45 s)	2.2 mM	106 bp
	rs9315400 (C/T)	C allele: GCAGCCTAGCATTTTACATC T allele: GCAGCCTAGCATTTTACATT COM: GTCTCTTTTTCGCAACCTTG	55°C (45 s)	2.5 mM	100 bp
	rs877098 (C/T)	C allele: AAGGGAGCTTGGAAATGAAGTC T allele: AAGGGAGCTTGGAAATGAAGTT COM: GAGGTGGAGCTTGCAGTGAG	56°C (1 min)	2.5 mM	227 bp
	rs945369 (A/C)	A allele: GTCCCTGTTTTTAGAAAAAAAGAA C allele: GTCCCTGTTTTTAGAAAAAAAGAC COM: GGAAGTAAACAGATCAGGGAG	59°C (1 min)	2.5 mM	187 bp
	rs7333214 (T/G)	T allele: AACATTTATCCAGGAATTGATATT G allele: AACATTTATCCAGGAATTGATATG COM: CAAATTTGCCAACAGACAATGC	57°C (1 min)	2.5 mM	230 bp

		Controls primers			

*AMELX *		CTLA: CCCACCTTCCCCTCTCTCCAGGCAAATGGG CTLB: GGGCCTCAGTCCCAACATGGCTAAGAGGTG			360 bp

COM: common primer (reverse). ∗The mismatches of the allele-specific primers are underlined. SNP ID: identification of the SNP. AMELX: human amelogenin gene used as an internal amplification control.

**Table 4:** Hardy-Weinberg proportions in the groups studied.

Gene	SNP	HWE *P* value (CTL)	HWE *P* value (patients)
*GJB2 *	rs3751385	0.143	**0.001**
	rs7994748	0.711	6.692 × 10^−11^
	rs7329857	0.994	0.615
	rs7987302	0.917	0.895

*GJB6 *	rs7322538	0.740	0.435
	rs9315400	0.653	0.557
	rs877098	4.312 × 10^−18^	3.221 × 10^−27^
	rs945369	**0.0082**	**0.003**
	rs7333214	0.995	0.156

HWE: Hardy-Weinberg equilibrium test was done using Pearson's goodness of fit *χ* ^2^ test and *P* value <0.05 was considered to show significant deviation of the observed genotypes from Hardy-Weinberg proportions. Significant deviations values from Hardy-Weinberg equilibrium are shown in boldface.

**Table 5:** Comparative analysis between minor allele frequency described in the database and minor allele frequency in the whole study group.

Gene	SNP ID (nucleotide change)	MAF ref	MAF study	*P* value (ref × study)
*GJB2 *	rs3751385 (C/T)	T = 0.360	0.405	0.513
	rs7994748 C/T	C = 0.481	0.173	3.442 × 10^−6^
	rs7329857 C/T	T = 0.079	0.043	0.287
	rs7987302 G/A	A = 0.098	0.026	**0.035**

*GJB6 *	rs7322538 G/A	A = 0.214	0.114	0.056
	rs9315400 C/T	T = 0.362	0.451	0.200
	rs877098 C/T	T = 0.429	0.486	0.418
	rs945369 A/C	C = 0.352	0.400	0.472
	rs7333214 T/G	T = 0.302	0.236	0.293

MAF (ref) ≥0.05: minor allele frequency information from public database, NCBI dbSNP Build 132; MAF (study): minor allele frequency in the whole study group. Significant values are shown in boldface.

**Table 6:** Association analysis of selected SNPs in *GJB2* and *GJB6* genes with the ARNSHL.

Gene	SNP	Samples	Allele distribution		*P*/*P* ^*^ corrected	OR (95% CI)	Genotype distribution			*P* value
			1	2			1 1	1 2	2 2	
*GJB2 *	rs3751385	CTL	198 (0.75)	66 (0.25)	1.12 × 10^−13^/1.011 × 10^−12^	4.04 (2.77–5.89)	70 (0.53)	58 (0.44)	4 (0.03)	5.83 × 10^−13^
	1 = C 2 = *T *	Patients	104 (0.43)	*140 (0.57) *			32 (0.26)	40 (0.33)	50 (0.41)	
	rs7994748	CTL	58 (0.22)	206 (0.78)	**0.004/0.036**	2.01 (1.25–3.25)	8 (0.06)	42 (0.32)	82 (0.62)	1.91 × 10^−5^
	1 = C 2 = *T *	Patients	30 (0.12)	*214 (0.88) *			10 (0.08)	10 (0.08)	102 (0.84)	
	rs7329857	CTL	262 (0.99)	2 (0.01)	3.86 × 10^−5^/3.48 × 10^−4^	11.70 (2.70–50.61)	130 (0.98)	2 (0.015)	0 (0.00)	2.53 × 10^−5^
	1 = C 2 = *T *	Patients	224 (0.92)	*20 (0.08) *			102 (0.84)	20 (0.16)	0 (0.00)	
	rs7987302	CTL	261 (0.99)	3 (0.01)	**0.035/**0.315	3.72 (1.01–13.68)	129 (0.98)	3 (0.02)	0 (0.00)	**0.0323**
	1 = G 2 = *A *	Patients	234 (0.96)	*10 (0.04) *			112 (0.92)	10 (0.08)	0 (0.00)	

*GJB6 *	rs7322538	CTL	232 (0.88)	32 (0.12)	0.704	0.87 (0.50–1.50)	101 (0.76)	30 (0.23)	1 (0.01)	0.764
	1 = G 2 = A	Patients	218 (0.89)	26 (0.11)			96 (0.79)	26 (0.21)	0 (0.00)	
	rs9315400	CTL	154 (0.58)	110 (0.42)	0.108	1.33 (0.94–1.89)	43 (0.33)	68 (0.51)	21 (0.16)	0.129
	1 = C 2 = T	Patients	125 (0.51)	119 (0.49)			35 (0.29)	55 (0.45)	32 (0.26)	
	rs877098	CTL	139 (0.53)	125 (0.47)	0.336	1.19 (0.84–1.69)	11 (0.08)	117 (0.89)	4 (0.03)	**0.0006**
	1 = C 2 = T	Patients	122 (0.50)	122 (0.50)			0 (0.00)	122 (1.0)	0 (0.00)	
	rs945369	CTL	145 (0.55)	119 (0.45)	**0.0144/**0.126	0.64 (0.45–0.92)	31 (0.23)	83 (0.63)	18 (0.14)	1.5334 × 10^−6^
	1 = *A* 2 = C	Patients	*160 (0.65) *	84 (0.35)			61 (0.50)	38 (0.31)	23 (0.19)	
	rs7333214	CTL	45 (0.17)	219 (0.83)	**0.0003/0.0027**	0.46 (0.30–0.70)	4 (0.03)	37 (0.28)	91 (0.69)	**0.0004**
	1 = *T* 2 = G	Patients	*75 (0.31) *	169 (0.69)			7 (0.06)	61 (0.50)	54 (0.44)	

CI: confidence interval; odds ratio (OR) between groups was determined by logistic regression. *P*: *P* value calculated by chi-squared test or Fisher's exact test for difference in allele and genotype frequency between cases and controls. ^*^ *P* value after Bonferroni's correction. The allele frequency statistically significant is shown in italic. Significant values are shown in boldface (*P* value <0.05).
